# Repurposing of FDA-approved drugs against cancer – focus on metastasis

**DOI:** 10.18632/aging.100941

**Published:** 2016-04-02

**Authors:** Béla Ozsvári, Rebecca Lamb, Michael P. Lisanti

**Affiliations:** The Breast Cancer Now Research Unit, Institute of Cancer Sciences, CRUK Manchester Institute, University of Manchester, UK

**Keywords:** tumor metastasis, repurposing, antibiotics, aspirin

Research on existing FDA-approved drugs is increasing in the field of anti-cancer drug development. Drug repurposing may allow progressing through the drug development process more quickly as well as reducing project costs compared to traditional *de novo* drug discovery. One of the main reasons why a non-cancer drug may prove effective against cancer is that not all of its biological targets may have been identified when the drug was originally developed. Until recently, the main focus in oncology drug development has been on the tumor cell — first with traditional chemotherapy and then with more targeted agents. But much of the focus has now shifted to targeting the tumor micro-environment, especially its key role in metastasis and drug resistance.

**Figure 1:**
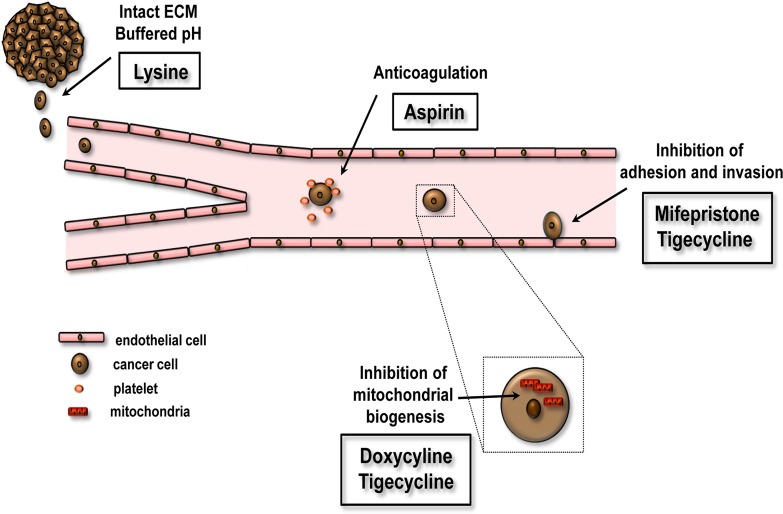
Effects of FDA-approved non-cancer drugs on early events of tumor metastasis Lysine buffers the acidic extracellular pH and increases the integrity of the extracellular matrix (ECM). Aspirin inhibits platelet activation and thrombocytosis. Doxycycline and tigecycline has been shown to inhibit mitochondrial biogenesis. Mifepristone or tigecycline suppresses cell migration and invasion.

Wan L. et al. proposed that initiation of circulating tumor cell adhesion to the local vascular endothelium is an important first step for circulating tumor cells to start the metastatic cascade. They showed that a combination of 3 FDA-approved drugs, the over-the-counter drug aspirin, the well-known antibiotic doxycycline and mifepristone (a progesterone receptor antagonist known as an abortifacient pill) used together with the amino acid lysine could effectively and safely prevent cancer metastasis (Figure 1.) [1]. This quadruplet drug combination has successfully inhibited adhesion of mouse melanoma (B16-F10) and human melanoma (M619) cell lines to either endothelial cells or extracellular matrix via down-regulating cell adhesion molecules ICAM-1 and α4-integrin. The drug treatment also inhibited platelet-cloaked aggregation of tumor cells in a dose-dependent manner. In their *in vivo* experiment, a four-day pre-treatment followed by a 30-day oral administration of the quadruplet drug combination to mice inoculated with melanoma cells produced significant inhibition of cancer metastasis in the lung dose-dependently without any marked side effects. In addition, the authors confirmed that all the four of these drugs were required for inhibition of cancer metastasis.

Aspirin, a widely used anti-inflammatory drug, a potent COX-2 inhibitor and an antiaggregant has been recently shown that its regular intake might reduce long-term risk of colorectal, oesophageal, gastric, biliary, and breast cancers and the risk of distant metastasis as well [2]. Add-Aspirin, the world's largest ever phase III clinical trial was launched in the UK in October 2015. Oncologists are looking to assess whether regular aspirin use after standard therapy prevents recurrence and prolongs survival in patients with non-metastatic common solid tumors (breast, colorectal, gastro-oesophageal, and prostate) [3].

We proposed a novel strategy for the treatment of primary tumors and advanced metastases via the selective targeting of cancer stem-like cells and identified a conserved phenotypic weak point - a strict dependence on mitochondrial biogenesis. Given that mitochondria have a bacterial origin, antibiotics also target mitochondrial translation and impair mitochondrial function. In our study we have shown that treatment with 4-5 different classes of FDA-approved antibiotics, especially doxycycline can be used to eradicate cancer stem-like cells in several tumor types (Figure 1) [4].

Pulvino et al. describe in their paper that a connectivity map (a collection of genome-wide gene expression profiles of cultured human cells treated with various bioactive small molecules) analysis uncovered several tetracycline antibiotics. Among them doxycycline as the most potent inhibitor of NF-κB signaling. Also, doxycycline successfully inhibited the growth of diffuse large B-cell lymphoma *in vitro* and in a mouse xenograft model. Importantly, this effect may be achievable in human sera with a therapeutic dose of the drug [5].

Hu et al. showed that tigecycline, another tetracycline antibiotic, suppressed cell proliferation, cell invasion and migration in melanoma (Figure 1). It induced cell cycle arrest at G0/G1 phase through the downregulation of p21 promoted cell cycle progression via CDK2/cyclin E cell kinases. In addition, intraperitoneal treatment with tigecycline significantly reduced tumor growth *in vivo* in a subcutaneous model of two human melanoma cell lines (A375 and MV3) in nude mice [6].

Cancer is a leading cause of death worldwide and despite the investment of millions of dollars of large pharmaceutical companies and governments into anti-cancer drug development, the current chemotherapeutic approaches are highly costly while their efficacy is not universally effective in all patients. These factors are leading to repurposing of well-known FDA-approved drugs, which can bypass the early stages of drug development, even Phase I clinical trials in some cases, thus reducing project costs and time. Taken together, the previously mentioned studies show that FDA approved drugs can be successfully applied in tumor therapies.
